# Using TMS-EEG to assess the effects of neuromodulation techniques: a narrative review

**DOI:** 10.3389/fnhum.2023.1247104

**Published:** 2023-08-14

**Authors:** Alessandro Cruciani, Marco Mancuso, Valerio Sveva, Davide Maccarrone, Antonio Todisco, Francesco Motolese, Francesca Santoro, Fabio Pilato, Danny Adrian Spampinato, Lorenzo Rocchi, Vincenzo Di Lazzaro, Fioravante Capone

**Affiliations:** ^1^Department of Medicine and Surgery, Unit of Neurology, Neurophysiology, Neurobiology, and Psychiatry, Università Campus Bio-Medico di Roma, Rome, Italy; ^2^Fondazione Policlinico Universitario Campus Bio-Medico, Rome, Italy; ^3^Department of Human Neurosciences, Sapienza University of Rome, Rome, Italy; ^4^Department of Anatomical and Histological Sciences, Legal Medicine and Orthopedics, Sapienza University, Rome, Italy; ^5^Department of Medical Sciences and Public Health, University of Cagliari, Cagliari, Italy

**Keywords:** TMS-EEG, neuromodulation, transcranial evoked potentials, electroencephalography analysis, motor evoked potentials

## Abstract

Over the past decades, among all the non-invasive brain stimulation (NIBS) techniques, those aiming for neuromodulatory protocols have gained special attention. The traditional neurophysiological outcome to estimate the neuromodulatory effect is the motor evoked potential (MEP), the impact of NIBS techniques is commonly estimated as the change in MEP amplitude. This approach has several limitations: first, the use of MEP limits the evaluation of stimulation to the motor cortex excluding all the other brain areas. Second, MEP is an indirect measure of brain activity and is influenced by several factors. To overcome these limitations several studies have used new outcomes to measure brain changes after neuromodulation techniques with the concurrent use of transcranial magnetic stimulation (TMS) and electroencephalogram (EEG). In the present review, we examine studies that use TMS-EEG before and after a single session of neuromodulatory TMS. Then, we focused our literature research on the description of the different metrics derived from TMS-EEG to measure the effect of neuromodulation.

## 1. Introduction

**FIGURE 1:**
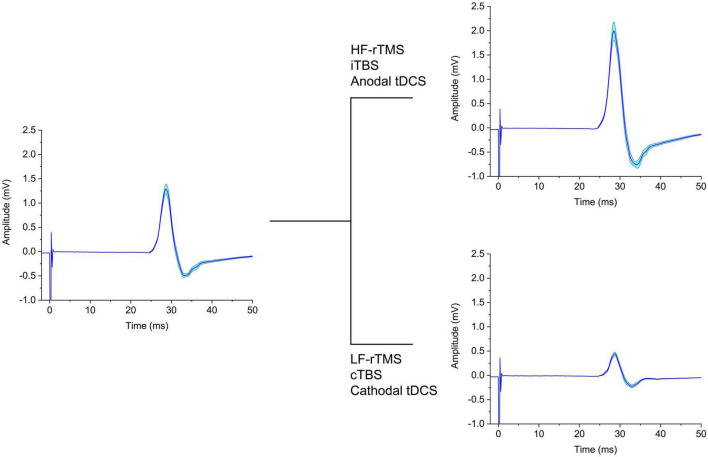
Schematic representation of the effect on MEP of different neuromodulatory techniques. The left panel depicts baseline MEPs, which are either increased **(upper right panel)** or decreased **(lower right panel)** in amplitude by the NIBS protocols indicated. tDCS, transcranial direct current stimulation; cTBS, continuous theta-burst stimulation; iTBS, intermittent theta-burst stimulation; HF-rTMS, high-frequency repetitive transcranial magnetic stimulation; LF-rTMS, low-frequency repetitive transcranial magnetic stimulation.

The term neuromodulation indicates long-lasting changes in synaptic efficacy, that persists after stimulation (Latorre et al., 2019; Lefaucheur et al., 2020). Over the past decades, neuromodulatory non-invasive brain stimulation (NIBS) techniques have gained a high level of interest due to their ease of application, lack of major side effects and translational value for therapeutic applications (Chase et al., 2020; Lefaucheur et al., 2020; Morone et al., 2022). The most common are transcranial direct current stimulation (tDCS) (Chase et al., 2020; Morone et al., 2022), transcranial alternating current stimulation (tACS) (Antal and Paulus, 2013; Ibáñez et al., 2022) and repetitive transcranial magnetic stimulation (rTMS) (Lefaucheur et al., 2020). Neuromodulation is often assessed by changes in motor evoked potential (MEP) amplitude, whereby its increase or decrease indicates corresponding changes to the excitability of the primary motor cortex (M1). However, given the multiple elements which play a role in MEP generation and modulation, interpreting a seemingly simple measure as its amplitude may not be straightforward (Spampinato et al., 2023). Additional factors, such as the contribution of spinal excitability, and the high inter-subject variability of input/output relationship may bias the information provided by MEP (Darling et al., 2006; Rossini et al., 2015; Rawji et al., 2020). The changes in MEP amplitude, limited to either increase or decrease, have possibly led to a simplistic, dichotomous interpretation of the effects of different neuromodulation techniques with very distinct underlying mechanisms (Figure 1). For instance, anodal tDCS (AtDCS), high-frequency rTMS and intermittent theta-burst stimulation (iTBS) (Di Lazzaro et al., 2011; Cirillo et al., 2017) all lead to increase in MEP amplitude, while continuous theta-burst stimulation (cTBS), low-frequency rTMS and cathodal tDCS are thought to cause MEP amplitude reduction (Di Lazzaro et al., 2011; Cirillo et al., 2017). By coupling TMS and electroencephalography (TMS-EEG) it may be possible to obtain more detailed information on cortical responses to external stimuli, compared to MEP (Chung et al., 2015; Hernandez-Pavon et al., 2023). Despite technical challenges (Mancuso et al., 2021; Cristofari et al., 2023), TMS-EEG allows the assessment of cortical function unbiased by spinal excitability and provides a complex signal from which many different variables can be calculated. These include measures in the time and time/frequency domain, such as TMS-evoked potentials (TEPs), Global Mean Field Potential (GMFP), local mean field potential (LMFP), TMS-related spectral perturbation (TRSP), and inter-trial phase clustering (ITPC) (Rocchi et al., 2018; Biondi et al., 2022), which can be used to assess local cortical responses to stimulation or activity and connectivity in distributed brain networks (Casula et al., 2020; Raffin et al., 2020). These measures have different physiological basis and reflect diverse aspects of cortical dynamics; therefore, it is plausible that their combination may yield information on the mechanisms of action of neuromodulation protocols additional to those provided by MEP amplitude changes alone. However, the effects of different NIBS are only seldom compared and, therefore, such information is not readily available. In the present work, we reviewed studies that used TMS-EEG to assess the effect of neuromodulation protocols (Table 1). We focused on the cortical areas more frequently investigated, especially M1 and the dorsolateral prefrontal cortex (DLPFC), comparing the effects of different protocols on various TMS-EEG measures to get more insight on the effects of neuromodulatory NIBS techniques.

**TABLE 1:** Articles that used TMS-EEG as a final readout of neuromodulatory NIBS techniques.

References	Technique	Metrics	Studied population	Meaning
Casula et al., 2014	I: 1 Hz rTMS-M1 II: 1 Hz rTMS-V1	MEPs, TEPs, LFMP	I:15 HS (7 F) II: 15 HS (7 F)	1 Hz rTMS-M1: ↓ MEPs, ↑ P60 and N100 TEPs (↑ inhibition GABAb-mediated post-synaptic potentials), ↑ LMFP 86–216 ms in the ipsilateral hemisphere
Zhou et al., 2022	1 Hz rTMS-M1 in 3 groups: -AAA (*n* = 22); -PPP (*n* = 10); -APA (*n* = 15)	MEPs, TEPs	46 HS	AAA vs. APA, N40 ↑ trend in AAA vs. PPP, ↓ P60 in AAA and PPP vs. APA, ↑ N100 in AAA vs. APA and PPP
Bai et al., 2021	iTBS-M1, iTBS-SMA, iTBS-V1	TEPs, GMFP, ISP, TMS-induced Oscillations	18 HS (6 F)	iTBS-M1: TEPs–N100 ↓ in bilateral hemispheres (contralateral > ipsilateral); early α-band synchronization in left-posterior, central and right-anterior cortex, ↓ GMFP in N100 time window (115 ms, 120–121 ms)
Gedankien et al., 2017	iTBS-M1	MEPs, TEPs, GMFP, LFMP	17 HS (9 F)	N15-P30 amplitude = , MEPs ↑, GMFP = , LFMP =
Vernet et al., 2013	cTBS-M1	MEPs, TEPs, TMS-induced EEG synchronization, eyes-closed resting EEG	10 HS (4 F)	↓ MEPs, ↑θ-band power and ↓β-band power in eyes-closed resting EEG, ↓θ- and α-band power and ↑β-band power in single TMS pulse
Ding et al., 2022	iTBS-M1 IH: - 11 Active iTBS; - 11 Sham iTBS.	TEPs, LMFP, GMFP, ERSP, TMS-evoked Oscillatory Response	22 SP	LMFP = , GMFP = , TMS-evoked Oscillatory Response = , Natural Frequency ↓ at baseline in the IH, ↑ after iTBS in the IH
Noh et al., 2012	cTBS-left M1: I group: Sham; II group: Active	MEPs, TEPs, LFMP, GFMP, TRSP	26 HS (13 F)	↑ in θ- and β-frequency in TRSP
Rocchi et al., 2018	cTBs-M1: I: 1 mV MEP PA-AP-LM coil orientation; II: 50% max individual MEP amplitude with the same coil orientation	MEPs, TEPs, resting EEG power, LMFP, TRSP, ITPC	13 HS (5 F)	↓ ITPC in the δ-, θ- and γ-band; ↓ TRSP in the α- and θ- band and ↑ TRSP in high β-range, ↓ LMFP
Desideri et al., 2018	100 Hz rTMS-M1 ipsilateral and contralateral, synchronized to the negative peak of the ongoing sensorimotor μ-rhythm	Resting-state EEG, TEPs, TMS-induced Oscillations, LMFP	23 HS	TEPs = , ↑ N100 LMFP 30 min after rTMS, ↓ P180 LMFP of the phase × time-dependent
Esser et al., 2006	5 Hz rTMS -M1	GMFP	7 HS	↑ GMFP in 18–55 ms; ↑ TMS-evoked activity in the prefrontal area
Pellicciari et al., 2013	Anodal vs. Cathodal tDCS-M1 (ipsilateral and contralateral)	TEPs, LMFP and TMS-evoked oscillatory activity	16 HS (8F)	Oscillatory brain activity = , ↑δ- and α-band power at both stimulation polarities
Chung et al., 2017	Sham vs. cTBS vs. iTBS DLPFC	TEPs, LICI, TMS-evoked oscillations	10 HS (4 F)	↑ N100 and P200 during iTBS, ↑ LICI and TMS-evoked θ oscillations during iTBS, ↓ LICI and TMS-evoked θ oscillations during cTBS
Chung et al., 2018	50%, 75% and 100% RMT -iTBS Prefrontal Cortex	TEPs, TMS-evoked oscillations	16 HS	↑ N100 in 50% and 75% RMT post 5-min (75>50% RMT), ↑ TMS-evoked θ oscillations during iTBS in ipsilateral fronto-central area and γ oscillations in ipsilateral parieto-occipital area
Luo et al., 2023	iTBS–DLPFC	TEPs	32 HS	↓ P200
Zrenner et al., 2020	iTBS over the negative phase of the individual α-peak	TEPs	22 MDD	TEPs = , ↑ in N100 and P200 with non-α locked iTBS
Desforges et al., 2022	iTBS–DLPFC	TEPs, SICI, LICI	14 HS	↓ P30, ↑ N45, ↓ N100 and ↑ P200, ↓ SICI- and LICI-induced inhibition on P30 and P200 TEPs amplitude, ↓ TMS-induced oscillations in θ
Dhami et al., 2021	iTBS–left DLPFC; cTBS–right DLPFC	TEPs	16 MDD; 16 HS	↑ N45, N100 and P200 =
Coyle et al., 2023	iTBS–DLPFC	TEPs	30 mTBI; 28 HS	↓ N45, N100 =
Gordon et al., 2018	tDCS: anode- left DLPFC; cathode–right DLPFC	TEPs	22 HS	↓ TEP amplitude 90–200 ms
Hill et al., 2017	High-definition-tDCS or bipolar-tDCS in DLPFC	TEPs, TMS-evoked oscillations	19 HS	↑ P60 5-min bipolar-tDCS and 30-min high-definition-tDCS, ↓ N100, ↓ TMS-induced oscillations in β and γ in parieto-occipital area 30-min after high-definition-tDCS
Hill et al., 2018	High-definition-tDCS; - left DLPFC ± PC	TEPs, TMS-evoked oscillatory power	22 HS	↑ P60 in T2 (post-30 min) in DLPFC after hd-tDCS; ↓ N100 in T2 in DLPFC + PC after hd-tDCS
Ye et al., 2022	10 Hz rTMS – DLPFC	TEPs	34 HS	↓ Ipsilateral positive and contralateral negative N120, ↓ in left insular EEG activity and ↑ right frontal EEG activity
Gordon et al., 2022	100 Hz rTMS synchronized in phase, anti-phase or random to the frontal θ-rhythm oscillation	TEPs	22 HS	TEPs =
Xia et al., 2019	10 Hz rTMS – DLPFC	TEPs, Eigenvalues, S-Estimators, Mutual Information Exchange, GMFP	14 HS; 21 DOC	↑ Global effective connectivity in HS and MCS contrarily to VS
Pellicciari et al., 2017	iTBS - left DLPFC; cTBS – right DLPFC	TMS-evoked oscillations	1 MDD	↑ P60 over left frontal area and ↓ over right parietal area, ↓ N100 across frontal, central, parietal and fronto-central regions
Bai et al., 2016	10 Hz rTMS- DLPFC	TEPs, PCI, GMFP	1 DOC; 5 HS	↑ GMFP in 30–100 and 200–400 ms time windows, ↑ PCI
Bai et al., 2017	10 Hz rTMS – DLPFC	GMFP, LMFP	16 DOC: 9 VS; 7 MCS	↑ GMFP in 0–100 ms and 100–200 ms in MCS; ↑ in 0–100 ms and ↓ in 300–400 ms in VS
Koch et al., 2018	rTMS – Precuneus	TEPs, GMFP, TMS-evoked oscillatory activity	14 AD	↑ TMS-evoked oscillatory activity in 60–90 ms and ↑β-band activity over parietal area
Koch et al., 2022	real or sham rTMS – Precuneus	TEPs, GMFP, TMS-evoked oscillatory activity	50 AD	TEPs 10–40 ms and 90–130 ms = , ↑ TMS-evoked oscillatory activity in fast γ-band
Liu et al., 2022	Active vs. Sham 40 Hz rTMS bilateral angular gyrus	PSD- resting state EEG	37 AD; 41 HS	↑γ-Oscillation power
Song et al., 2019	1 Hz rTMS – right PPC	GMFP, LMFP	20 PI (8 F); 20 HS (8 F)	↑ Connections in the frontal mid-line and left posterior temporal areas, ↓ connections in the right central and right temporal region
Romero Lauro et al., 2014	Active vs. Sham Anodal tDCS-PPC	TEPs, LMFP, GMFP	14 HS	↑ GMFP during and after active anodal tDCS in 0–100 ms, ↑ LMFP at the end of stimulation in Parietal and Frontal clusters bilaterally
Grasso et al., 2021	Anodal tDCS-PPC	TEPs, ERPs	32 HS (16F)	↑ ERPs P2 component, TEPs =
Pisoni et al., 2018	I: anodal tDCS over the LIFG and TMS over the left BA6; II: sham tDCS and TMS over the left BA6; III: anodal tDCS over the LIFG and TMS over the left PPC	GMFP, LMFP, global and local SCD	18 (10 F)	TEPs, GMFP and LMFP = in BA7, significant changes in the middle-latency component of GMFP and in the early-latency component of LMFP over C1 and C2

HS, healthy subjects; SP, stroke patients; ISP, interhemispheric signal propagation; AAA, active stimulation with the same coil for test-treat-test phases; PPP, realistic placebo coil stimulation for all three phases; APA, active coil stimulation for tests and placebo coil stimulation for treatment; TRSP, TMS-related spectral perturbation; ERSP, event-related spectral perturbation; ITPC, inter-trial phase clustering; IH, ipsilesional hemisphere; PPC, posterior parietal cortex; AD, Alzheimer’s disease; PSD, power spectral density; LIFG, left inferior frontal gyrus; SCD, significant current density; PI, primary insomnia; DOC, disorder of consciousness; PCI, perturbation complexity index; VS, vegetative state; MCS, mild conscious state; MDD, major depressive disorder; mTBI, mild traumatic brain injury; PC, parietal cortex.

### 1.1. Methods

We selected only articles where TMS-EEG were conducted at baseline and then repeated after the delivery of a single session of one neuromodulatory NIBS protocol. Keywords used for our search, either alone or in combination, were the following: “TMS-EEG”; “neuromodulation”; “electroencephalography”; “tDCS”; “tACS”; “rTMS”; “iTBS”; “cTBS”. Several databases were used, including Pubmed, Web of Science, Embase, and Google Scholar. Since most of the literature targeted two specific areas (i.e., M1 and DLPFC), they are discussed in more detail, while other, less studied cortical areas are discussed in a following section.

### 1.2. Neuromodulation techniques

**FIGURE 2:**
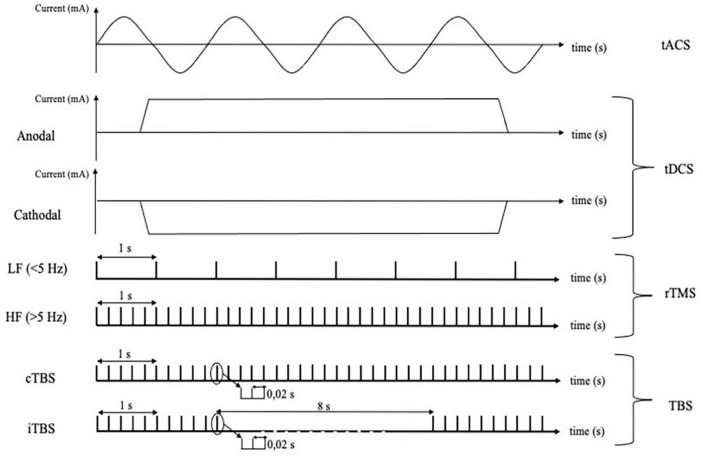
Different techniques of cortical non-invasive neuromodulation and relative protocols using electrical or magnetic stimulation. tACS, transcranial alternating current stimulation; tDCS, transcranial direct current stimulation; rTMS, repetitive transcranial magnetic stimulation; LF, low frequency; HF, high frequency; TBS, theta burst stimulation; cTBS, continuous theta burst stimulation; iTBS, intermittent theta burst stimulation.

Transcranial magnetic stimulation and electroencephalography has been used to assess the effects of several non-invasive neuromodulation techniques, including tACS, tDCS, rTMS, iTBS and cTBS (Figure 2). tDCS involves the administration of a constant current and is often referred to as anodal or cathodal, based on the electrodes closer to the stimulation site. It is often assumed that anodal tDCS shifts membrane potential toward more positive values, thus increasing neuronal excitability and spontaneous activity, while the opposite would occur for cathodal stimulation (Nitsche and Paulus, 2000; Nitsche et al., 2008, 2005). In fact, the net cortical effects are complex and depend on a number of factors which are difficult to quantify, including cell orientation and different sensitivity of neuronal compartments to exogenous currents (Liu et al., 2018). Differently, tACS entails the use of alternate current oscillating at a given frequency (Liu et al., 2018). This type of stimulation is thought not to change the overall neuronal firing rate, but rather to cause temporal biasing of action potentials and entrainment of activity in local and more distributed neuronal networks (Krause et al., 2019; Johnson et al., 2020). Instead of using direct electrical stimulation of the cortex, rTMS involves the administration of repetitive trains of subthreshold magnetic pulses, organized in regular or patterned fashion (Jannati et al., 2023) which induce a secondary electric field in the underlying neural tissue (Hallett, 2007). High-frequency (>5 Hz) rTMS is thought to induce excitatory effects on the cortex, while low-frequency (<1 Hz) stimulation is considered inhibitory. Another neuromodulation technique is patterned rTMS, the most common being theta-burst stimulation, where pulses are delivered in 5 Hz bursts, with pulses at 50 Hz frequency. Whereas iTBS generally increases cortical excitability, cTBS is thought to have opposite effects (Huang et al., 2005). Despite the very different mechanisms of action and immediate effects, the neuromodulation protocols mentioned above seem to be able to induce long-lasting effects on cortical excitability via similar plasticity mechanisms, including long-term potentiation (LTP) and long-term depression (LTD) like effects (Jannati et al., 2023). For these reasons, neuromodulatory techniques might be exploited as therapeutical and monitoring tools in several neurological conditions (Lefaucheur et al., 2020; Motolese et al., 2023, 2022).

### 1.3. TMS-EEG metrics

**FIGURE 3:**
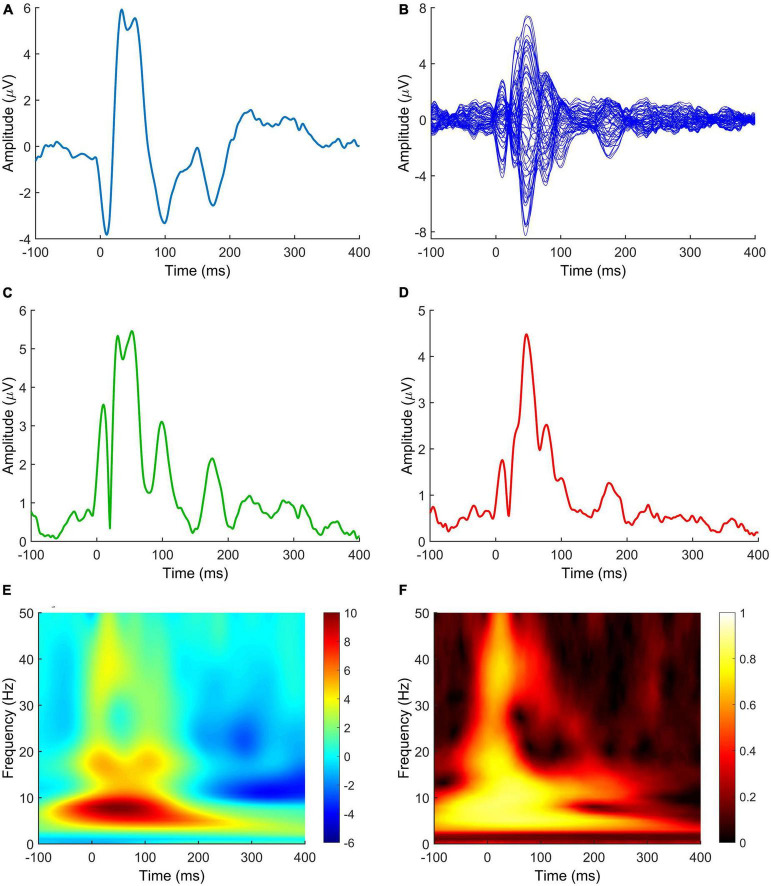
TMS-EEG metrics measuring the effect of neuromodulation after NIBS protocols. **(A)** TEP; **(B)** butterfly plot; **(C)** LMFP; **(D)** GMFP; **(E)** TRSP; **(F)** ITPC. Signals in panels **(A,E,F)** are derived from C3 electrode. The LMFP in panel **(C)** is calculated from C3, C1, CP3, and CP1 electrodes. Spectrograms in panels **(E,F)** are obtained by convolution via Morlet Wavelets, with 1 ms temporal resolution and 1 Hz frequency resolution. Data are taken from a previously published paper (Mancuso et al., 2021). TEP, TMS-evoked potential; LMFP, local mean field potential; GMFP, global mean field potential; TRSP, TMS-related spectral perturbation; ITPC, inter-trial phase clustering.

The complexity of the field is further compounded by the fact that different metrics can be derived from TMS-EEG to measure the effect of neuromodulation (Figure 3). Here follows a short review of those most found in the literature.

Transcranial evoked potentials (TEPs) are the most intuitive measure of TMS-EEG activity, consisting of the time domain signal evoked by TMS averaged across trials. Their temporal and spatial profile depends on the site of stimulation and intensity used, but standardized responses can be evoked from well-established sites of stimulation, such as M1 and DLPFC (Massimini et al., 2005; Tremblay et al., 2019; Leodori et al., 2021; Rocchi et al., 2021). Although they present different issues in interpretation (Conde et al., 2019; Rocchi et al., 2021), they have shown to be reliable markers of local cortical excitability (Premoli et al., 2019; Leodori et al., 2021) and may provide a useful tool to study cortico-cortical (Bortoletto et al., 2015) and cortico-subcortical (Leodori et al., 2022, 2021) connectivity.

The LMFP and GMFP are time domain measures related to the TEP. The LMFP is usually calculated as square root of the squared TEP averaged across a limited number of contiguous electrodes which define a region of interest (ROI); as such, the LMFP provides a measure of the strength of local cortical activation following a TMS pulse (Casula et al., 2018; Rocchi et al., 2018). Differently, the GMFP is calculated as the time-varying standard deviation of the signal obtained by the whole set of recording electrodes (Lehmann and Skrandies, 1980) and provides information about activation across the whole scalp, where signal averaging would not yield meaningful information due to phase cancellation (Fecchio et al., 2017; Leodori et al., 2023).

More advanced TMS-EEG metrics are calculated in the time/frequency, rather than time domain, and require signal decomposition by one of several signal processing solutions (i.e., convolution with complex Morlet wavelets). The TRSP allows to quantify changes in power at discrete frequencies following the TMS pulse, compared to a baseline segment. ITPC (sometimes referred to as inter-trial coherence, ITC) is a measure of consistency of phase angles across multiple trials and therefore gives information about the strength of phase-locking induced by TMS (Delorme and Makeig, 2004). TRSP and ITPC provide complementary information and using both is recommended to capture different aspects of cortical activation induced by TMS (Kallioniemi and Daskalakis, 2022).

Finally, Perturbation Complexity Index (PCI) is operationally defined as the normalized complexity index of the spatiotemporal pattern of cortical activation triggered by a direct TMS perturbation. This measure expresses the level of joint presence of integration and segregation in the human brain and has been shown to provide key information on consciousness (Casali et al., 2013).

## 2. Primary motor cortex (M1)

### 2.1. TEPs

Among the various TMS-EEG parameters to assess how M1 responds to neuromodulation, TEPs are the most reported outcome measure. Over this brain region, it is important to consider that single-pulse TMS typically evokes four distinct components in the recorded EEG signal: two positive peak at a latency of 30 (P30) and 60 ms (P60), and two negative peak at 45 ms (N45) and 100 ms (N100). In the context of rTMS protocols, which can induce after-effects on cortical excitability, two influential studies have investigated how TEPs are modulated following low-frequency rTMS (Casula et al., 2014; Zhou et al., 2022). In the first study, Casula et al. (2014) delivered 1 Hz rTMS over the left M1 to 15 healthy volunteers (HV) and observed increased amplitudes of the P60 and N100 components in the TEPs. Notably, the N100 component has been suggested to reflect inhibitory processes and was found to be particularly affected by rTMS, indicating a modulation of GABAergic inhibition (Casula et al., 2014; Premoli et al., 2014). Supporting this idea, a larger study with 46 participants following the same protocol replicated the increase in the N100 component but reported opposite results for the P60 component, showing a decrease in amplitude (Zhou et al., 2022). The authors argued that the divergent findings are likely due to the larger sample size and methodological differences in TMS settings between the two studies (Zhou et al., 2022). Zhou et al. (2022) used 200 pulses and a stimulation intensity of 110% of the resting motor threshold (RMT) to improve signal-to-noise ratio, while Casula et al. (2014) used a stimulation intensity of 120% RMT and 50 pulses. The choice of using suprathreshold intensities in both studies may explain the opposite results regarding the P60, as this early component could be influenced by peripheral re-afferent motor activation linked to the TMS stimulation intensities (Petrichella et al., 2017). Also, the effects of TBS on TEPs have been investigated with divergent results. Bai et al. (2021) found a significant reduction in the amplitude of the N100 component after iTBS in 18 healthy subjects, while Gedankien et al. (2017) did not observe any differences in TEPs amplitude in a study with a similar sample size. It is important to note that an analytical difference between the two studies may partially explain these discrepant findings. Gedankien et al. (2017) did not analyze late TEPs components, including the N100. Their decision to neglect this analysis is due to a lack of appropriate noise masking procedure, which could result in contamination of late TEP components by auditory evoked potentials (Mäki and Ilmoniemi, 2010; Rocchi et al., 2021). Regarding cTBS, only one study has investigated the modulation of TEPs following stimulation (Vernet et al., 2013). The authors found an inhibition of the P30, consistent with a positive correlation between decreased P30 and MEPs and a negative correlation between increased N44 and MEPs, as described in other studies (Mäki and Ilmoniemi, 2010; Ferreri et al., 2011). Moreover, cTBS also increased TMS-evoked theta and alpha oscillations, while it decreased beta oscillations, suggesting a widespread effect of neuromodulation on networks functioning.

### 2.2. Oscillatory analysis

Transcranial magnetic stimulation and electroencephalography studies investigating oscillatory cortical activity have provided valuable insights into the effects of neuromodulation on M1, albeit findings thus far have been inconsistent. For example, Bai et al. (2021) conducted a study with 18 healthy participants and found that iTBS to M1 significantly decreased α-band frequencies in the TMS-induced oscillatory activity. This effect was observed in specific cortical areas, namely the left-posterior, central, and right-anterior cortical areas. Importantly, the authors demonstrated the specificity of these results to M1 stimulation, as there was no changes in oscillatory activity were iTBS was applying to the supplementary motor area (SMA) or primary visual cortex. In contrast, Ding et al. (2022) investigated a group of 22 subacute stroke patients and did not find differences in terms of TMS-evoked oscillatory power amplitude following M1 iTBS. However, they did observe an increase in the maximum frequency power from θ-band to early β-band in the ipsilesional M1, which they attributed to enhanced fast thalamocortical-driven oscillatory activity. It should be noted that this study lacked a control group, which limits the interpretation of these findings. Regarding M1 continuous theta-burst stimulation, several studies have explored its effects on local oscillatory activity in healthy subjects (Noh et al., 2012; Vernet et al., 2013; Rocchi et al., 2018). Specifically, Rocchi et al. (2018) found that cTBS led to a significant decrease in δ-, θ-, and γ-frequency bands when using a TMS stimulation intensity that elicited a half maximum individual MEP amplitude. Additionally, Rocchi et al. (2018) reported a reduction in intra-trial phase synchrony (ITPS) in the δ- and θ-frequency range, suggesting increased neural “noise” in the stimulated region and disrupted oscillatory synchronicity induced by TMS pulses. In another study by Vernet et al. (2013), cTBS over M1 resulted in decreased activity in θ- and α-frequency bands and increased activity in the high-β range. However, the authors recorded a relatively small number of TEPs at different time points after cTBS, which may not have been sufficient for obtaining reliable measurements due to the low signal-to-noise ratio of TEPs. These differences in results across studies may be attributed to the number of TMS pulses administered for TMS-EEG outcome measures. In contrast, Noh et al. (2012) observed an increase in θ- and β-frequency TRSP, but these results are limited by a technical factor: TRSP was averaged across the entire epoch segment after TMS, which introduced a significant amount of noise into the analysis. Therefore, the interpretation of these findings should be approached with caution. Additionally, Desideri et al. (2018) examined the effects of applying 200 bursts of 100 Hz rTMS in different pre-defined brain states, specifically the positive and negative peaks of the local μ-rhythm. In a sample of 23 healthy individuals, the authors found no evidence of changes in cortical excitability as measured by TEPs or alterations in oscillatory power. In summary, TMS-EEG studies investigating oscillatory cortical activity following neuromodulation in M1 have produced mixed results. While some studies have reported changes in specific frequency bands and localized cortical areas, others have failed to find significant effects. Methodological factors, such as the choice of stimulation parameters and the number of TMS pulses administered, may contribute to the discrepancies observed across studies.

### 2.3. GMFP and LMFP

To date, only few studies explored the effects of rTMS on global TMS-evoked EEG activity, using GMFP as the main outcome measure. Esser et al. (2006) conducted the first study in this area, demonstrating a specific positive modulation of GMFP between 18 and 55 ms following 5 Hz rTMS administration in 7 healthy volunteers. This modulation indicated an increase in TMS-evoked activity bilaterally in the premotor cortex, suggesting that the effects of rTMS on M1 rapidly dissipate while persisting activation occurs in the premotor areas (Esser et al., 2006). Furthermore, Bai et al. (2021) recently conducted a study involving 18 HV and found that a single session of M1 intermittent theta-burst stimulation significantly decreased GMFP within the time window of the N100 component (115 ms, 120–121 ms). In contrast, Gedankien et al. (2017) observed no statistically significant changes in GMFP following 1 Hz rTMS in 17 healthy older volunteers, and Ding et al. (2022) reported no significant alterations in GMFP after 50 Hz rTMS in the ipsilesional M1 of 22 stroke survivors. These discrepancies could be attributed to several factors, including differences in the mean age of the studied population, sample size, and stimulation frequency utilized. In terms of the local mean field power, many studies have assessed the aftereffects of both magnetic and electric neuromodulation protocols. Casula et al. (2014) investigated the effects of a single session of 1 Hz rTMS on M1 in 15 healthy individuals and observed a robust increase in the late LMFP component (86 ms to 216 ms). Notably, this effect was localized to the stimulated hemisphere, suggesting a positive modulation of local GABAergic circuitry. Conversely, Rocchi et al. (2018) observed an opposite effect on LMFP following cTBS applied to the cortex. Interestingly, this effect occurred without any evidence of modulation in MEPs, indicating that TMS-EEG may be more sensitive in assessing the effects of neuromodulatory techniques. Pellicciari et al. (2013) evaluated the impact of anodal and cathodal transcranial direct current stimulation on TEP, LMFP, and oscillatory activity. The results suggested a polarity-specific modulation of the initial and later components of LMFP, reflecting complex patterns of direct and indirect cortical activations or inhibitions within the motor circuitry, possibly related to modifications in synaptic efficacy within the cortex itself. Similarly, Gedankien et al. (2017) reported no significant modulation of LMFP at any individual time point following iTBS. Ding et al. (2022) also observed no significant change in LMFP after iTBS applied to the ipsilesional M1 of 22 stroke survivors, consistent with their findings for GMFP. However, a study by Desideri et al. (2018) demonstrated a modulation of LMFP following high-frequency (100 Hz) excitatory rTMS synchronized to the negative and positive peaks of the EEG μ-rhythm. Specifically, they observed a phase-dependent increase in the N100 LMFP during stimulation synchronized to the positive μ peak, and a phase-dependent decrease in the positive peak at 180 ms (P180) LMFP during stimulation synchronized to the negative μ peak. In summary, investigations into the effects of rTMS on global TMS-evoked EEG activity have yielded mixed results. Some studies have shown positive modulation of GMFP in premotor areas following rTMS to M1, indicating persistent activation in these regions. Positive and negative modulations of LMFP have been observed, suggesting complex patterns of cortical activations or inhibitions within the motor circuitry. These discrepancies may be attributed to variations in population characteristics, stimulation parameters, and outcome measures. Overall, further research is needed to better understand the effects of rTMS on TMS-evoked EEG activity and its implications for cortical excitability and oscillatory power.

## 3. DLPFC

### 3.1. TEPs

We identified different studies using rTMS, tDCS, TBS or tACS that used DLPFC TEPs as markers of neuromodulation. Regarding TBS, most of the evidence supports an effect on late (N100-P200) TEP components. In a study on 10 HV, local TEPs were assessed after TBS and sham stimulation, delivered at 80% RMT, on the left DLPFC. The N100 and P200 were increased after iTBS, but not after cTBS. Nevertheless, a P200 increase was also obtained after sham stimulation (Chung et al., 2017). The same authors found that the N100 and N200 modulation was maximized when iTBS was applied at 75% RMT intensity (Chung et al., 2018). On the other hand, another group has tried to probe the effect of iTBS over the same area in HVs showed no modulation of the N100 with a decrease in P200 amplitude, measuring effects 30 min after the stimulation (Luo et al., 2023). The null effect on the N100 is compatible with the observation described by Chung et al. (2017), that the effects of iTBS on this component disappeared 30 min after stimulation. Conversely, the effect on the P200 component resulted difficult to explain. In an attempt to refine TBS paradigms, Zrenner et al. (2020) delivered iTBS on the negative phase of the individual alpha rhythm in 22 patients affected by Major Depressive Disorder (MDD), but did not find any modulation of the TEP. Surprisingly, an increase in N100 and P200 amplitude was found when non-alpha locked iTBS was delivered. Despite the interestingly results those evidences resulted in a difficult interpretation since no noise masking was applied and no HV control group was available to compare normal effects of iTBS. In contrast with the evidence reported above, a reduction in N100 and P200 amplitude was shown after iTBS in a study involving 14 HV, but again no noise masking was applied (Desforges et al., 2022), hindering again the interpretation of results (Rocchi et al., 2021). Similarly, Dhami et al. (2021) found no clear N100 or P200 modulation in 16 HV and 16 patients with MDD after iTBS or cTBS over the left DLPFC; as previously, results are difficult to interpret as only earplugs were used to suppress the TMS click. Additionally, TEPs were obtained after iTBS was applied five out of 7 days for 2 weeks, without specifying the time between the last application of iTBS and TEPs; therefore, a N100 modulation similar to shown previously (Chung et al., 2017) might have been missed. Another study involving 28 HV and 30 patients with mild traumatic brain injury (mTBI) found no evidence of N100 modulation after iTBS, here using proper noise masking and recording TMS-EEG signal within 5 min from TBS (Coyle et al., 2023). A possible explanation of the lack of positive results might be the use of a particular statistical method for scalp EEG analysis, the TANOVA (Topographic analysis of variance), which is imbalanced toward topographic rather than amplitude differences across scalp maps and may lack sensitivity for the N100 modulation. We cannot also exclude that mTBI causes subtle changes in connectivity and a diffuse and transient synaptic dysfunction. Earlier TEP components (P30 and N45) have been shown to be modulated by iTBS as well, with contrasting results. For example, a study on 14 HV found a decrease of the P30 and an increase of the N45 after iTBS (Desforges et al., 2022). By contrast, another study found a reduction in N45 amplitude after iTBS in HV and mTBI patients, although this modulation was not consistent over different time points (Coyle et al., 2023). The role of TMS-EEG as a marker for the effects of iTBS has been explored also considering more complex metrics than local TEPs. For example, a study involving 16 HV and 16 MDD found that bilateral TBS stimulation over DLPFC (iTBS over the left, cTBS over the right) increased the N45 amplitude after TMS stimulation of the right inferior parietal lobule (IPL), a change which correlated with the right DLPFC-right IPL connectivity at rest, and which was found to be represented at the source level by a decrease in right insular and IPL activity (Dhami et al., 2021). Another neuromodulation technique often coupled with TMS-EEG is tDCS. Further, most of the evidence revolves around the N100-P200 complex. Gordon et al. (2018) applied tDCS on 22 HV with 1.5 mA intensity and a montage consisting in anode on the left DLPFC and cathode on the right DLPFC. They found an amplitude reduction of TEPs between 90 and 200 ms after stimulation over the left prefrontal cortex. Hill et al. (2017) applied high-definition tDCS (hd-tDCS) (a five elctrode tDCS that allows more focal stimulation) either alone or coupled with a working memory task (n back) over the left DLPFC of 19 HV. The main founding was a reduction of the N100 evoked from TMS stimulation over the left DLPFC. This decrease was greater when tDCS was coupled with the task and was positively correlated with the behavioral performance. The same stimulation pattern induced an increase in P60 amplitude in the same study and in another involving 20 HV in two different states (at rest, or performing a cognitive task engaging working memory), but no behavioral correlate was identified. The authors concluded that the relative modulation of P60 and N100 induced by tDCS reflected a change in excitatory/inhibitory balance (Hill et al., 2017). Another interesting neuromodulatory technique tested in several TMS-EEG study is the rTMS. Ye et al. (2022) documented that 10 Hz rTMS delivered over the left DLPFC in 34 HV reduced the ipsilateral positive and contralateral negative N120 component, which, at the source level, was attributed to a decrease in left insular activity and increase in right frontal activity. However, according to the site of stimulation and with the correlation with pain threshold, it is possible that the observed N120 component might be caused by activation of the secondary somatosensory cortex (García-Larrea et al., 1995; Rocchi et al., 2016). By contrast, no clear TEP modulation was found by Gordon et al. (2022) after 100 Hz rTMS delivered in phase, antiphase or randomly with respect to frontal theta oscillations in 22 HV. A different approach has been used in 14 HV and 21 patients with disorders of consciousness (DOC) by Xia et al. (2019). The authors found no differences in TEPs and GMFA after 10 Hz rTMS modulation over the DLPFC in DOC patients. Conversely, significant changes were observed in the HV group in GMFA after the same stimulation protocol. Moreover, an increase in global connectivity was observed, as Max Eigenvalue of the evoked connectivity matrix significantly increased after 10 Hz rTMS treatment among HV and S-estimator values of the same matrix increased in HV and minimally conscious state (Xia et al., 2019).

### 3.2. Oscillatory analysis

Some studies have focused on the effect of neuromodulation on changes in brain oscillatory activity assessed by TMS-EEG. Most of the evidence derives from studies involving TBS. In a study on 10 HV, left DLPFC iTBS, compared to cTBS, induced an increase in theta band TRSP in the contralateral prefrontal cortex and an increased suppression of theta power following paired pulse stimulation with an interstimulus interval compatible with long intracortical inhibition. This increased suppression was positively correlated with the increase in amplitude of the N120, hinting that iTBS increased cortical inhibitory tone (Chung et al., 2017). The same authors, in a second study involving the application of iTBS and sham stimulation over the DLPFC in 16 HV, found that real stimulation led to an increase in theta power in the ipsilateral fronto-central area at rest, an increase in alpha power in the ipsilateral parietal area during a following 2 n-back task in the letter presentation period, and an increase in gamma power in the ipsilateral parieto-occipital cortex in a following 3 n-back task during the maintenance period (Chung et al., 2018). Interestingly, the alpha band modulation was positively correlated with the reaction time at the 3 n-back test. Notwithstanding the similarity of stimulation parameters between studies (both sharing single pulse stimulation at 120% RMT and similar iTBS protocol intensities, i.e., 75% and 80% RMT), the two studies only partially reproduced the same results: while the former (Chung et al., 2017) found an increase in the stimulation ROI (F3, FC3, F1, FC1) theta band after iTBS, when extending the analysis at the whole scalp level, no significant oscillation modulation was identified, in contrast with the modulation in theta found in the latter (Chung et al., 2018). This difference might be attributed to an increase in sample size in the second study (16 vs. 10 subjects), a notion corroborated by the fact that the ROI in which theta power was found to be increased in the first study is similar to the cluster of electrodes in which theta was found to be increased in the second (assuming higher sensitivity for a ROI level analysis). On the other hand, iTBS-induced TRSP modulation in the alpha and gamma range during specific memory tasks, but not at rest, suggests a complicate relation between TMS-EEG measures and network state during recording, which warrants a deeper understanding of the coupling between TMS-EEG and behavioral tasks. On the other hand, when focusing on TMS induced (i.e., non-phase locked) oscillations, a study performed on 14 HV found an opposite effect, with reduction of theta power and of theta paired-pulse suppression over the stimulated area (Desforges et al., 2022). The discrepancies between findings are hard to reconcile and might be due to different topographical analysis (ROI vs. scalp-wide) and methodology of TRSP calculation, since Desforges et al. (2022) analyzed non-phase locked oscillations by subtracting the TEP from the TRSP, while Chung et al. (2018) analyzed the TRSP without any subtractions. Finally, an interesting approach is that proposed by Pellicciari et al. (2017), who, in line with the theory of imbalanced frontal alpha oscillations in MDD, tested bilateral DLPFC TMS-EEG after delivering sequential iTBS over the left DLPFC and cTBS over the right DLPFC in a patient affected by MDD. They found that left DLPFC iTBS decreased theta and alpha band oscillations and increased power in faster frequencies, while right DLPFC cTBS increased alpha oscillatory activity (Pellicciari et al., 2017). Albeit this study suffers from a limited sample size, being a case report, the concordance between the neurophysiological background of MDD and the observed response is relevant. DLPFC tDCS has been used less than TBS, with a TMS-EEG study on 20 HV showing a reduction in theta and gamma TRSP in the frontal and parietal area (Hill et al., 2017) and another one involving 22 HV showing that hd-tDCS over the left DLPFC can induce a reduction of the occipital beta and gamma power (Hill et al., 2018). rTMS delivered on 22 HV at 10 Hz on the DLPFC, at the negative phase of local alpha oscillations, increased TRSP in the alpha and theta frequency bands locally and in the high gamma band in the contralateral parieto-occipital cortex, together with an increase in fronto-central theta gamma coupling, while positive peak phase rTMS reduced frontal ipsilateral TRSP in theta and high beta ranges (Gordon et al., 2022). This evidence suggests that TRSP shows some sensitivity to neuromodulation, but little agreement is found between studies, making a definite interpretation difficult.

### 3.3. GMFP and LMFP

Different studies have investigated the effects of transcranial stimulation on the DLPFC to evaluate changes in GMFP in subjects suffering from vegetative state (VS) or minimally conscious state (MCS). Bai et al. (2016) evaluated a 10 Hz rTMS protocol, delivered for 20 daily consecutive sessions on the left DLPFC of a single MCS patient. Baseline GMFP evaluation of the subject showed a reduction in cortical excitability in temporal windows of 30–100 and 200–400 ms compared to five age matched female healthy volunteers (Bai et al., 2016). After a single session, in the absence of clear clinical improvement, the patient’s GMFP increased in the same temporal window after stimulation. After 20 days of rTMS, the GMFP increased to a level similar to HV, in the presence of a clinical improvement. In a further work, the same group investigated the effects of 20 min of anodal tDCS on the left DLPC of 9 VS and 7 MCS subjects (Bai et al., 2017). They found an increase in GFMP in 0–100 and 100–200 ms time frames in MCS subjects and between in 0–100 and 300–400 ms time windows in VS subjects. 14 VS, 7 MCS and 14 HV received a single session of 10 Hz rTMS over the left DLPFC in a study by Xia et al. (2019). They found a non-significant trend toward an increase of GMFP among MCS subjects in 0–100, 100–200 and 200–300 ms time windows. A single study investigated the effects on LMFP by rTMS of the left DLPFC. Bai et al. (2017) found a significant increase in LMFP between 0 and 100 ms in frontal, left hemispheric and central areas, and between 100 and 200 ms in frontal and right hemispheric areas in MCS subjects. A significant increase was likewise observed in the 0–100 ms time window (left hemisphere), whereas a significant reduction in excitability was observed during 300–400 ms (frontal and left hemisphere areas), in VS subjects.

### 3.4. PCI

Only one study assessed the effect of a neuromodulatory technique on PCI (Bai et al., 2016). In this study, Bai et al. (2016) investigated PCI and coma recovery scale revised (CRS-R) in a patient with MCS following brain hemorrhage before and after 20 sessions of 10 Hz rTMS over left DLPFC. CRS-R score increased from 8 to 30, paralleled by an increase in PCI from 0.28 to 0.37 and a clinical improvement from MCS- to MCS+ (Bai et al., 2016).

## 4. Other cortical areas

### 4.1. PC

The first study utilizing TMS-EEG to investigate the effects of neuromodulation applied to the precuneus (PC) was conducted by Koch et al. (2018), a sham-controlled study that evaluated cognitive (episodic memory) and cortical changes (TEP, GMFP, TRSP) following a 2-week session of high-frequency rTMS in 14 patients with Alzheimer’s disease (AD). A daily stimulation session consisted of 1,600 stimuli applied at 20 Hz over the PC with a stimulation intensity set at 100% RMT. Following the completion of the 2-week protocol, a notable improvement in episodic memory was observed in the real condition, as compared to sham stimulation. Additionally, TMS-EEG results showed increased GMFP amplitude in medial frontoparietal areas and increases in PC beta oscillatory activity in the real condition (Koch et al., 2018). These results suggest that high-frequency rTMS over the PC results in the modification of functional connections between the precuneus and medial frontal areas within the Default Mode Network (DMN) – a critical neural network which involvement occurs early during the AD continuum - and that stimulation increases the engagement of the PC. In a more recent study by Koch et al. (2022), the authors conducted a randomized, double-blind, sham-controlled study in which AD patients received either real (25 patients) or sham rTMS (25 patients) over the precuneus. This study involved a 24-week treatment, consisting of a 2-week intensive course where rTMS (or sham) was administered to the precuneus five times per week, followed by a 22-week maintenance phase with weekly stimulation. During the real treatment, each rTMS session comprised 40 trains of 2-second duration delivered at 20 Hz, with 28-s intervals between trains, resulting in 1,600 stimuli. As in the previous work, the authors assessed clinical dementia scores and TMS-EEG measures of excitability and oscillatory activity corresponding to the precuneus region (TEP, TRSP) at different stages of the treatment. Here, the authors found that patients given PC stimulation displayed stable performance of dementia clinical scores and TEP amplitudes. In contrast, patients treated with sham showed worse clinical scores and a significant reduction in TEP amplitudes in time windows between 10 to 40 ms and from 90 to 130 ms after 24 weeks. Moreover, the authors show that patients given real rTMS displayed increases in fast gamma oscillations (31–48 Hz) over the PC, a result not found in patients given sham rTMS. Furthermore, the changes in TEPs were correlated with changes in visual perception and attention, providing further evidence for the utility of TEPs in the precuneus as a biomarker for neuromodulation (Koch et al., 2022).

### 4.2. Parietal cortex

The parietal cortex has also received considerable consideration as an area in which TMS-EEG has been used to explore the effects of neuromodulation. Liu et al. (2022) conducted a sham-controlled study that targeted the angular gyrus (AG) bilaterally with high-frequency rTMS in 37 patients with probable AD. Here, patients received treatment every other day, three times a week for 4 weeks (12 treatment days). Each session included 30 rTMS trials, alternating between left AG and right AG (15 trials each), with pulses applied at 40 Hz with 40% MSO. Each trial consisted of a 2-s pulse train with a 58-s interval, with a total of 2,400 stimulus pulses delivered. To study the effects of rTMS on dynamic effective functional connectivity between the angular gyrus and other brain regions, the authors analyzed TMS-EEG data within the 3–80 Hz frequency range to gain insights into the neural network interactions following rTMS. Dynamic connectivity was assessed using a time-varying adaptive multivariate autoregressive (AMVAR) model and an adaptive directed transfer function (ADTF) (Zhang et al., 2017). Their findings revealed that rTMS strengthened connectivity between anterior and posterior brain regions. This increase in connectivity was associated with improved cognitive function, as indicated by increased scores in MoCA and MMSE (measuring general cognitive function) and decreases in ADAS-Cog scores (a clinical measure used to assess the severity of dementia). These positive effects were observed immediately after stimulation and persisted at the 8-week follow-up. In contrast, the sham group did not exhibit significant changes in cognitive function or connectivity measures. Furthermore, Liu et al. (2022) observed a reduction in grey matter volume loss through functional magnetic resonance imaging (fMRI) analysis and increased gamma oscillation power after active rTMS treatment at electrode T5 and Cz, suggesting beneficial effects on neural activity. In a study conducted by Song et al. (2019), low frequency (1 Hz) rTMS was applied to the right posterior parietal cortex (r-PPC, P4 electrode) in 20 patients with primary insomnia and 20 HV. The treatment consisted of 14 daily sessions and the authors compared the network activity between the two groups using ADTF. Compared to HV at baseline, the analysis revealed abnormal connectivity patterns in patients, characterized by excessive outflow between the frontal mid-line and left posterior temporal regions and a diminished outflow between the right central and right temporal regions. Interestingly, following the intervention, patient abnormal connectivity patterns were restored, and the changes were correlated with improvement in insomnia symptoms. Romero Lauro et al. (2014) conducted a TMS-EEG study in 14 HV to investigate the effects of real and sham anodal tDCS stimulation on the right PPC. For real AtDCS application, a constant current of 0.75 mA was applied for 15 min with an 8-s fade-in/fade-out period, while sham tDCS involved the same arrangement and stimulation parameters, but the stimulator was turned off after 30 s. The authors observed that AtDCS increased the GMFP during and after stimulation (0–100 ms) and increased early TEPs (0–50 ms) in the targeted right PPC, as well as in the contralateral homolog area and bilaterally in frontal regions. Later TEP components (100–150 ms) displayed increased activity on-line in the right temporal area, while no changes were observed in the sham stimulation. Similarly, Grasso et al. (2021) similarly investigated the effects of AtDCS on visuo-spatial contextual learning (VSCL) in 32 healthy subjects. Although changes in ERPs (enhanced P2 component of cortical response) and in task performance after AtDCS were observed, no changes were found in TEPs when compared to sham stimulation. The group receiving AtDCS did exhibit a tendency toward a sustained early cortical response when stimulation and task execution were combined. Pisoni et al. (2018) examined the impact of real and sham anodal AtDCS on the left inferior frontal gyrus (LIFG) while recording TEPs from the left premotor cortex (a region involved in verbal fluency) and the left superior parietal lab (a control site). The authors found no significant changes in TEPs in the left superior parietal lobule following AtDCS. However, TEP measures in the left premotor cortex exhibited significant changes in middle-latency GMFP and early changes in LMFP over EEG electrodes C1 and C2. No significant effects were observed during sham stimulation, and no changes were found in late-latency components across groups. In a similar study by the same group, cathodal tDCS was applied to the right posterior parietal cortex while recording TEPs from the left posterior parietal cortex. Here, no significant changes in GMFP and LMFP were observed. Overall, these studies provide valuable insights into the effects of different types of transcranial direct current stimulation (tDCS) on TEPs and neural activity in the parietal cortex, highlighting the complex and region-specific nature of tDCS modulation.

## 5. Discussion and conclusion

In the present review, we investigated the role of TMS-EEG to assess the effects of the most common non-invasive neuromodulation techniques, with the aim of gaining insight into the mechanism of action of NIBS, as well as reviewing the reliability of TMS-EEG studies in evaluating NIBS effects. The most studied TMS-EEG variable are TEPs amplitude and TRSP, which are studied predominantly on M1 and DLPFC. Regarding TEPs, the N100 is the component which is most often affected by different NIBS techniques. Moreover, when we use TEPs as a readout of neuromodulatory techniques not all the effects that we generally see on MEPs are confirmed. Indeed, no evidence of modulation of the N100 comes from cTBS (Vernet et al., 2013) and high frequency rTMS, although few studies have been conducted. By contrast, iTBS increases the N100 amplitude when stimulating the DLPFC (Zrenner et al., 2020), while anodal tDCS decreases it (Hill et al., 2017). Based on those data we can assume that the effects of neuromodulatory techniques on MEPs cannot be completely applied on TEPs, especially for the N100 component. Nevertheless, real changes in the N100 are still difficult to assess, since it can be contaminated by saliency-related activity caused by the TMS click if the latter is not properly masked (ter Braack et al., 2015; Du et al., 2018; Conde et al., 2019; Rocchi et al., 2021), an issue not frequently assessed in the literature. This hinders the interpretation of some of the aforementioned TMS-EEG studies, which do not use effective masking of the TMS click, together with heterogeneity of stimulation parameters, time spans between neuromodulation and TMS-EEG, and statistical methods. This discrepancy between results on MEPs and TEPs also suggests a possibly differential effect of NIBS on different areas according to their different structure and physiology, an area still not thoroughly investigated. For instance it is well known that different brain areas has different natural frequency (Capilla et al., 2022), this could lead to possible bias in TMS-EEG oscillatory analysis. Another interesting but poorly studied correlations is between modulation of TMS-EEG metrics and behavioral variables (Pellicciari et al., 2013; Hill et al., 2017; Zrenner et al., 2020; Casula et al., 2022; Fong et al., 2023). This interestingly parameter could represent a feature which, if properly investigated, would greatly help to understand the mechanisms underlying NIBS and TMS-EEG responses. One possible methodology to minimize the technical discrepancy between studies is to use different analysis that provides complimentary information such as TRSP and ITPC. Nonetheless, in our literature review we have found only one study (Rocchi et al., 2018) that analyzed the same set of data with both. We hence stress the importance of such a practice to provide reliability to data interpretation. In the light of the great possibilities of TMS-EEG as a neurophysiological readout, the literature we gathered highlights a number of issues that limit interpretation of results. This is a promising field to study the effect of NIBS, since it would help discriminate the effect on different networks without being limited to local analysis. Moreover, using MEP as a readout of neuromodulatory techniques could result in excessive simplification of the neurophysiological outcome. Indeed, with MEP we can only assess changing in amplitude. This led to a simplistic dichotomy suffering from several bias (Spampinato et al., 2023). Coupling TMS with EEG we are able to obtain more complex and detailed information about cortical response to NIBS (Chung et al., 2015; Hernandez-Pavon et al., 2023). Nonetheless, these evaluations require considerable effort in standardization, artifact limitation, and rigorous methodological analysis, which, at the state of the art, does not seem to be matched by the literature (Massimini et al., 2005; ter Braack et al., 2015; Mancuso et al., 2021; Rocchi et al., 2021). This holds true especially when the technique is coupled with non-invasive neuromodulation, a field that already in itself comprises a plethora of technical and experimental variables, which are not always appropriately controlled by authors. However, there is clear need for effective biomarkers of neuromodulation efficacy. TMS-EEG might be a tool both for investigating the pathophysiology of neurological disorders – since many neurological conditions might be considered as neural networks disorders – and for monitoring the effects of neuromodulation when the latter is used for therapeutic purpose. In parallel, there is also the need of a more standardized approach for the clinical application of TMS-EEG. This is mandatory to make TMS-EEG a valuable tool not only for research but also for clinical application.

## Author contributions

AC, MM, VS, LR, DS, VD, and FC made substantial contributions to the conception or design of the work. AC, MM, VS, DM, AT, FS, FM, FP, DS, LR, VD, and FC drafted the work or critically revised it for important intellectual content, agreement to be accountable for all aspects of the work in ensuring that questions related to the accuracy or integrity of any part of the work are appropriately investigated and resolved, and approved the final version to be published.
